# Two Years of Growth Hormone Therapy in a Child with Severe Short Stature Due to Overlap Syndrome with a Novel SETD5 Gene Mutation: Case Report and Review of the Literature

**DOI:** 10.3390/genes16080859

**Published:** 2025-07-23

**Authors:** Giovanni Luppino, Malgorzata Wasniewska, Giorgia Pepe, Letteria Anna Morabito, Silvana Briuglia, Antonino Moschella, Francesca Franchina, Cecilia Lugarà, Tommaso Aversa, Domenico Corica

**Affiliations:** 1 Department of Human Pathology of Adulthood and Childhood, University of Messina, Via Consolare Valeria1, 98125 Messina, Italy; giovilup97@gmail.com (G.L.); giorgia.pepe@unime.it (G.P.); francesca.franchina8@gmail.com (F.F.); cecilialug@gmail.com (C.L.); tommaso.aversa@unime.it (T.A.); domenico.corica@unime.it (D.C.); 2 Pediatric Unit, AOU Policlinico G. Martino, Via Consolare Valeria 1, 98125 Messina, Italy; letteria.morabito@gmail.com; 3 Department of Biomedical, Dental, Morphological and Functional Imaging Sciences, University of Messina, 98100 Messina, Italy; silvana.briuglia@unime.it; 4 UOSD Genetica Medica, Grande Ospedale Metropolitano “Bianchi-Melacrino-Morelli”, 89100 Reggio Calabria, Italy; nimoschella@gmail.com

**Keywords:** Cornelia de Lange syndrome, growth hormone therapy, KBG syndrome, MDR23, *SETD5* gene mutation, short stature, overlap syndrome

## Abstract

Background: SET domain-containing 5 (SETD5) is a member of the protein lysine-methyltransferase family. *SETD5* gene mutations cause disorders of the epigenetic machinery which determinate phenotypic overlap characterized by several abnormalities. *SEDT5* gene variants have been described in patients with KBG and Cornelia de Lange (CdL) syndromes. Case description: A female patient with severe short stature and intellectual disability had been followed since she was 9 years old. Several causes of short stature were ruled out. At the age of 12 years, her height was 114 cm (−5.22 SDS), weight 19 kg (−5.88 SDS), BMI 14.6 kg/m^2^ (−2.26 SDS), and was Tanner stage 1. The target height for the proband was 151.65 cm (−1.80 SDS). The bone age (BA) was delayed by 3 years compared to chronological age. The growth rate was persistently deficient (<<2 SDS). Physical examination revealed dysmorphic features. Genetic analysis documented a de novo *SETD5* gene mutation (*c.890_891delTT*), responsible for phenotypes in the context of an overlap syndrome between the phenotype of MDR23, CdL and KBG syndromes. Recombinant growth hormone therapy (rhGH) was started at the age of 12 years. After both one year (+3.16 SDS) and two years (+2.9 SDS), the growth rate significantly increased compared with the pre-therapy period. Conclusion: This is the first case of a patient with overlap syndrome due to *SETD5* mutation treated with rhGH. The review of the scientific literature highlighted the clinical and molecular features of *SETD5* gene mutation and the use of rhGH therapy in patients suffering from CdL and KBG syndromes.

## 1. Introduction

Rare genetic disorders and/or syndromic conditions could be manifest as short stature, potentially associated with other clinical features [1]. Genetic causes of short stature can be documented through genetic testing, such as karyotyping, chromosome microarray, targeted gene sequencing, or exome sequencing [2]. Recombinant growth hormone (rhGH) therapy is indicated for patients with short stature caused by specific genetic disorders [3].

RhGH therapy has received approval from the United States Food and Drug Administration (FDA), European Medicines Agency (EMA), and Agenzia Italiana del Farmaco (AIFA) in a number of short stature conditions other than growth hormone deficiency (GHD), including syndromic and genetic diseases such as Turner syndrome (TS), Noonan syndrome (NS), Prader–Willi syndrome (PWS), and SHOX deficiency. Additionally, rhGH therapy is indicated also for other clinical disorders including children born small for gestational age (SGA) without checkup growth, chronic kidney disease, and idiopathic short stature (ISS) [4,5]. RhGH treatment improves growth outcomes and could also have several beneficial and positive effects on muscle tropism, body composition, and bone mineral density that might be compromised in syndromic conditions [6]. In addition, the efficacy of rhGH therapy in the growth pattern of patients with rare genetic syndromes, including Silver–Russel Syndrome, Kabuki Syndrome, and Duchenne muscular dystrophy, has been documented in the scientific literature even if rhGH treatment is indicated as off-label [7].

SET domain-containing 5 (SETD5) is a member of the protein lysine-methyltransferase family that regulates gene expression. *SETD5* gene (OMIM 615743) mutations cause disorders of the epigenetic machinery which determine phenotypic overlap characterized by several abnormalities. *SETD5* gene haploinsufficiency is found in specific conditions, such as mental retardation autosomal dominant 23 (MRD23) syndrome [8,9] or microdeletion of chromosome 3p25.3, characterized primarily by intellectual disability, neurodevelopmental disorders, and possible growth delay [8,9]. Recently, *SEDT5* gene variants have been described in patients with KBG syndrome (KBGS) and Cornelia de Lange syndromes (CdLS), genetic disorders characterized by short stature, delay in growth, and additional clinical features. In the literature, few reports are available on the use of rhGH in the KBG and Cornelia de Lange syndromes [10,11,12,13].

The objectives of this manuscript are to describe the first case of overlap syndrome with rhGH therapy and to carry out a review of the scientific literature on cases of KBG and CdL syndromes treated with rhGH.

## 2. Case Report

A 9-year-old Caucasian girl was referred for the first time to outpatient pediatric endocrinology due to short stature and deficient growth rate. She was born full term by non-consanguineous parents by cesarean section after a gestation complicated by gestational thrombophilia. According to Bertino’s charts, her birth weight and length were 3900 gr (−1.35 standard deviation score (SDS)) and 54 cm (−0.75 SDS), respectively, classified as adequate for gestational age. The neonatal period was uncomplicated. She started walking at 2 years of age and she showed severely delayed speech development (first words at the age of 3 years). From the age of two, due to a delay in psychomotor development and general muscle hypotonia, she practices rehabilitation therapy, including psychomotor skills and speech therapy.

The family history was negative for endocrinological diseases, and her parents were healthy. Her mother’s height was 148.2 cm, and her father’s height was 168.1 cm. The target height for the proband was 151.65 cm (−1.80 SDS).

**Figure 1:**
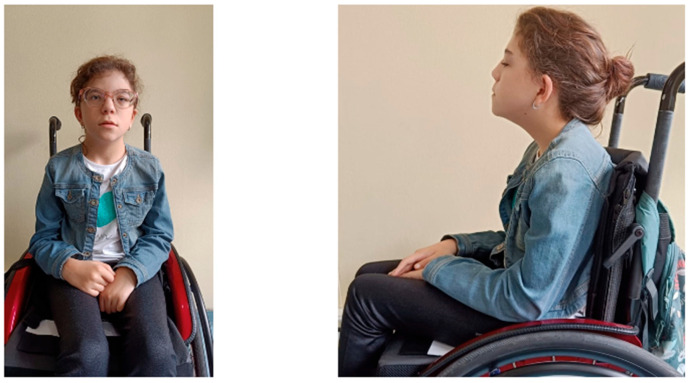
Images of the patient’s clinical dysmorphic features.

At the time of the first evaluation at the age of 9 years old, her height and weight were 103.3 cm (−5.46 SDS) and 15.1 kg (−5.08 SDS), respectively; her body mass index (BMI) was 14.18 kg/m^2^ (−1.71 SDS). She was prepubertal (Tanner stage 1). Bone age delay was estimated to be 2 years (with the Greulich and Pyle method). Physical examination revealed dysmorphic features, including a long triangular face, low-set and protruding ears, hypertelorism, a short nose with prominent columella and concave nasal ridge, a long philtrum, a thin upper lip, macrodontia of the central incisors, downturned corners of the mouth, pointed palate, and left palpebral ptosis. The child wears corrective lenses for myopia and astigmatism. No significant hearing loss or additional abnormalities were found. In addition, she presents an incomplete capacity to autonomy ambulation, and needs walking aids (Figure 1).

Biochemical examinations were performed. Endocrine workup showed adequate calcium-phosphorus homeostasis and thyroid function were normal. The screening for celiac disease was negative and hepatic and renal function were normal. Low serum insulin-like growth factor 1 (IGF-1) was detected (IGF-1:28, 4 ng/mL, −7.788 SDS sec Horenz 2021) [14] and it was assumed to be linked to the nutritional status of the patient. Evaluation of growth hormone secretion was performed with a clonidine stimulation test that documented a GH peak of 10.24 ng/mL [5]. The test showed a normal peak of circulating growth hormone (GH) (>8 ng/mL) according to Italian guidelines [5], so GHD was ruled out without the need for a second stimulation test. Echocardiography and abdominal ultrasound were normal.

**Figure 2:**
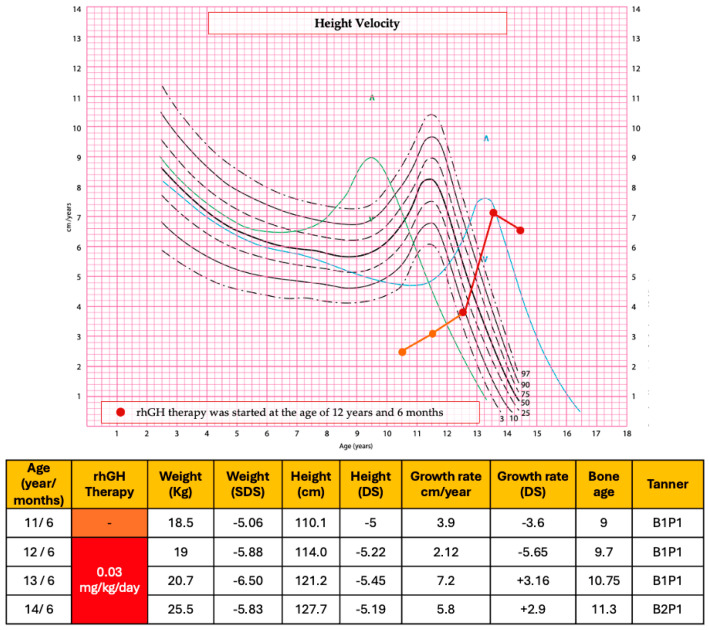
Auxological parameters and height velocity before and after the start of rhGH therapy. In the height velocity chart, the symbol ^ represents the 97th centile peak.

A six-monthly endocrinological and auxological follow-up was conducted with annual assessment of bone age (Figure 2). The patient’s growth has been slow and stunted for her age, as evidenced by the growth velocity of 3.9 cm/year (−3.6 DS).

In addition, neuropsychological counseling detected an intellectual disability with a total IQ of 48 (WISC-R) and brain MRI documented signs of hypomyelination at the level of the cerebral hemispheres, altered morphology of the basal nuclei, and atrophy of the cerebellum.

The karyotype analysis was normal, as well as array CGH. Exome sequencing detected a de novo *SETD5 gene* mutation (c.890_891delTT (p.Leu297fs*15)) classified as a pathogenic variant according to The American College of Medical Genetics and Genomics (ACMG). The patient’s phenotype is consistent with the SETD5 gene mutation in the context of an overlap syndrome between the phenotype of MDR23, KBG, and Cornelia de Lange syndromes.

Considering severe short stature and stunted statural growth, GH therapy was evaluated before the start of pubertal development as a possible off-label treatment to improve the growth outcome. Approval from a regional medical commission was obtained to prescribe off-label GH therapy for a patient with idiopathic short stature related to a rare and genetic mutation.

At the age of 12 years and 6 months [height 114 cm (−5.22 SDS), weight 19 kg (−5.88 SDS), Tanner stage 1] rhGH therapy was started at the dose of 0.03 mg/kg/day. Before the start of rhGH therapy, bone age was delayed by 3 years compared with chronological age and the final stature prediction was about 133 cm, severely lower than the target height of the proband. The patient showed excellent compliance with the treatment. No side effects or health complaints occurred during therapy.

After the first year of therapy, the growth rate had been 7.2 cm (+3.16 SDS) and her height was 121.2 cm (−5.45 SDS), her Tanner stage was B1P1, and her bone age was 10.7 years. IGF-1 levels during therapy were within normal limits.

After 2 years of treatment, at the age of 14 years and 6 months, the growth rate had been 5.8 cm/year (+2.9 SDS). Her height and weight were 127.7 cm (−5.19 SDS) and 25.5 kg (−5.83 SDS), respectively; BMI was 15, 64 kg/m^2^ (−2.51 SDS) and her Tanner stage was 2 (B2P1). Thelarche began when she was 13 years old. Bone age was progressed but still delayed compared with chronological age. The final stature prediction improved by approximately 6 cm compared to the pre-treatment assessment. Under rhGH treatment, the patient presented no notable clinical or laboratory adverse events; the glucose profile was normal in the different controls as well as IGF-1 levels.

Figure 2 summarizes the auxological data and growth velocity of the patient before and after the rhGH treatment.

The height velocity chart shows the range of normal year growth velocity for females according to age, through solid lines and dotted lines. The green and blue lines represent the early (+2 SDS) and late (−2 SDS) maturers, respectively. The patient’s growth has been slow and stunted for her age, as evidenced by the growth velocity (orange line) before the rhGH treatment. After the first year of therapy, the growth rate (red line) had been 7.2 cm (+3.16 SDS). After 2 years of treatment, at the age of 14 years and 6 months, the growth rate had been 5.8 cm/year (+2.9 SDS). The auxological data were evaluated referring to Cacciari and Tanner charts [15,16]. In the height velocity chart, the symbol ^ represents the 97th centile peak. 

## 3. Discussion

### 3.1. SETD5 Gene Mutation and Genetic Overlap Syndrome

SETD5 is a member of the protein lysine-methyltransferase family that regulates gene expression. The *SETD5* gene is located on 3p25.3, which codes for a multi-domain protein. *SETD5* is one of several genes implicated in epigenetic mechanisms. The SETD5 protein has specific domains (SET, H3K36, and PHD) that allow epigenetic changes to chromatin through the H3 methyltransferase activity [8]. The *SETD5* gene is mainly expressed in brain tissue, which is involved in neural progenitor proliferation and synapse development. *SETD5* expression is also elevated in other tissues, such as the intestine and the eye [17]. *SETD5* gene haploinsufficiency impairs brain development and neuronal function, causing neurodevelopmental disorders such as intellectual disability (ID) and autism spectrum disorders (ASD) [17,18,19]. Syndromic conditions related to the alteration of the *SETD5* gene are responsible for a wide clinical spectrum including neurodevelopment disorders. Additionally, the association between the disorders of epigenetic machinery and short stature have been investigated recently. As reported, *SETD5* is implicated in epigenetic modulation. Mendelian disorders of the epigenetic machinery (MDEMs) are caused by several genetic mutations and are characterized by largely phenotypic overlap with multiorgan abnormalities associated with short stature [20]. Thirty-three pathogenic/likely pathogenic variants in nineteen genes for epigenetic modulation were identified by whole-exome sequencing (WES) in a cohort of patients with short stature. On this occasion, a patient with short stature, a webbed neck, and a novel *SETD5* variant was reported for the first time [20].

Overlap syndromes are characterized by the occurrence in the same patient of two or more clinical findings of different diseases. *SETD5* gene mutations are described in different syndromic conditions, such as mental retardation autosomal dominant 23 (MRD23) syndrome, KBG syndrome, and CdL syndrome.

Mental retardation autosomal dominant 23 (MRD23) is a mendelian disorder of the epigenetic machinery [21,22]. Haploinsufficiency of the *SETD5* gene due to missense or punctiform mutation of the *SETD5* gene or the 3p25.3 microdeletion syndrome, which involves a chromosomal deletion that encompasses the *SETD5* locus, could cause clinical phenotypes related to MRD23 [8]. The clinical phenotype consists of ID, delayed growth, facial dysmorphisms, hypotonia, epilepsy, or EEG abnormalities. Other clinical phenotypes are renal abnormalities, syndactylism, gastrointestinal abnormalities, and scoliosis [23]. Growth retardation and short stature are detected in MRD23. In patients with a *SETD5* gene mutation, the growth pattern may be abnormal and different from the growth rate of patients with the microdeletion chromosome 3p25.3.

Patients with *SETD5* LoF variants typically present with normal head circumference, weight, and length at birth, and they are normocephalic. However, they may develop short stature during growth. Conversely, patients with microdeletion of chromosome 3p25.3 exhibit postnatal onset of microcephaly along with pronounced short stature [21,22,23,24]. Although short stature may be a clinical feature in patients with *SETD5* gene alterations, chromosome 3p deletions are associated with a more severe phenotype compared to *SETD5* LoF variants [25].

Chromatin-modifying disorders comprise an expanding group of neurodevelopmental disorders (NDDs) caused by mutations in functionally related chromatin genes. These syndromes are characterized by significant phenotypic overlap, as they share common clinical features, including intellectual disability, developmental delay, growth retardation, and a similar facial phenotype. Cornelia de Lange syndrome (CdLS) and KBG syndromes (KGBS) fall into this group [24].

CdLS (OMIM# #122470, #300590, #610759, #614701, #300882) is a genetic disease due to variants in cohesion genes including *NIPBL* (the most frequent gene mutated), *SMC1A*, *SMC3*, *RAD21*, and *HDAC8*. According to the 2018 consensus statement, classic CdLS is diagnosed if a patient presents a clinical score ≥11 points (Table 1) [24]. Recently, several genetic variants in non-cohesion genes have been found to cause a clinical phenotype of CdLS. Also, chromatinopathies and mutation in the methyltransferase’s gene are documented in patients with CdLS-overlapping features [26,27,28]. Parenti et al. described the first two patients with a clinical phenotype compatible with a mild form of CdLS and a genetic analysis positive to the SETD5 gene mutation, a de novo non-senso mutation (NM_001080517) and a microdeletion of chromosome 3p25.3, respectively [29]. Despite the fact that non-cohesion gene mutations may cause a clinical phenotype of CdLs, the average clinical score of patients in an *SETD5* cohort was statistically lower than the score of cases in an NIPBL gene mutated cohort. Thus, *SETD5* gene mutation can be considered responsible for a mild clinical form of CdLs [28].

KBGS (OMIM#148050) is a genetic disorder due to alterations of the chromatin-associated transcription machinery. KBGS is caused by haploinsufficiency of *ANKRD11* (ankyrin repeat domain-containing protein 11, OMIM*611192) gene, located on chromosome 16q24.3 [30], although the mutation is not detected in all patients with clinical features suggestive of KBGS [31]. Suggested diagnostic criteria are reported in Table 1 [30]. Crippa et al. described three patients with clinical diagnosis of KBGS associated with *SETD5* gene mutations, who were identified as having overlap syndromes based on common clinical features with *MRD23* and the genetic profile [32].

Clinical criteria for KBG and Cornelia del Lange Syndrome are reported by Moreal et al. [30] and by the 2018 consensus statement [26], respectively.

Our case presents a novel *SETD5* gene mutation (c.890_891delTT (p.Leu297fs*15)) and a heterogeneous clinical phenotype. The presence of *SETD5* mutation and the association of clinical features common to KBGS, CDLS, and MDR23 led to the diagnosis of overlap syndrome in our patient (Table 2). To the best of our knowledge, the scientific literature has not described patients with short stature more severe than −4 SD due to *SETD5* gene mutation. Our patient is the first case of *SETD5* gene mutation related to a severe form of growth delay with a height of −5.46 SDS at the age of 9 years old.

### 3.2. Focus on Growth Hormone Therapy and Growth Rate in CdL and KBG Syndromes

The scientific literature has reported few cases on the effects of recombinant human GH (rhGH) treatment in children with CdLS or KBGS (Table 3) [10,13]. There are no reports of rhGH treatment in patients with overlap syndrome.

De Graaf et al. reported the case of a girl born small for gestational age (SGA) with severe postnatal growth retardation who underwent rhGH treatment at age 4 years for failure to recover stature (height of −3.5 SDS). The diagnosis of CdLS was not made until age 10 years (de novo heterozygous mutation (c.771+1G>A) in the *NIPBL* gene). After 8 years of rhGH treatment, the patient had significant statural recovery of 1.8 SDS [10].

Short stature and postnatal growth delay are prevalent in KBG syndrome and spontaneous catch-up growth beyond childhood is limited. In a cohort study including 18 children and 14 adults with KBG syndrome, heights below –2.5 SDS was observed in 62% of the children and 36% of the adults [11]. Kang et al. [12] analyzed data from 77 individuals with *ANKRD11* aberrations (50 children and 22 adults) and short stature with heights below –2.0 SDS was present in 54% of the children and 68% of the adults [12].

A significant acceleration in height gain has been reported in children with KBG syndrome due to *ANKRD11* gene mutations treated with rhGH [11,12]. In one case, rhGH was administered due to a coexisting diagnosis of growth hormone deficiency (GHD). However, even in patients without GHD, rhGH treatment resulted in notable improvements in height.

For instance, a 10-year-old Caucasian boy with KBG syndrome caused by an *ANKRD11* frameshift mutation (p.Ser1279fs) presented with a height of −3.1 SDS and a bone age delayed by 3 years. After one year of rhGH treatment (35 μg/kg/day), he gained 0.6 SDS in height [11]. Similarly, a Chinese boy with a heterozygous point mutation (c.2579C>T) of the *ANKRD11* gene underwent GH replacement therapy for 24 months (0.15 units/kg/day), which resulted in a growth of 17.1 cm, corresponding to a statural recovery of 1.3 SDS [12].

An evaluation of growth outcomes in 13 children with KBG syndrome and *ANKRD11* haploinsufficiency, all with heights below the 3rd centile, revealed that 9 received rhGH therapy. Among these, 4 had confirmed GHD. All but one patient responded positively to treatment, although detailed growth rate data were unavailable for the non-responder. Overall, patients with KBG syndrome demonstrated a favorable response to rhGH therapy during the first year of treatment, especially when compared to four untreated KBG patients, who showed less significant growth progression [13].

In addition, therapy with rhGH has been found to be safe and effective in several long-term studies [33]. Available data in the scientific literature on the use of rhGH in KBG and CdL syndromes have reported no side effects during therapy, and no adverse effects were documented in our patient’s case either.

After two years of rhGH treatment, the statural outcome of our patient is satisfactory especially in terms of the statural growth rate. In fact, the latter has significantly improved since the first year of therapy and persisted to be satisfactory during the second year of therapy. The persistence of the significant height deficit compared with the average of female peers can be attributed not only to the syndromic condition but also to the delayed onset and slow progression of pubertal development. Moreover, the final stature prediction after the first two years of therapy was improved by about 6 cm compared with the pre-treatment assessment.

## 4. Conclusions

To the best of our knowledge, this is the first case of a patient with overlap syndrome due to *SETD5* mutation treated with rhGH. Variants of the *SETD5* gene have been recently classified as overlap syndrome due to the overlap of clinical features common to KBG, CdL, and MDR23 syndromes, as in the case of our patient. In these cases, growth hormone therapy might be effective not only in improving the growth rate during growth but also the final stature of these subjects, as reported in the few available cases of CdLS and KBGS treated with rhGH reported in the literature. Larger case series of patients are needed to evaluate the efficacy of rhGH therapy in this syndromic condition.

## Figures and Tables

**Table 1:** Diagnostic clinical criteria for KBG and Cornelia de Lange Syndromes.

**KBG syndrome (KBGS)** is characterized by **developmental delay/learning difficulties, speech delay, significant behavioral issue** and at least **two major criteria** or **one major and two minor criteria**	**Classic Cornelia de Lange syndrome (CdLS)** is defined with a **clinical score ≥ 11 points, of which at least 3 are cardinal features**. Non-classic CdLS presents a clinical score of 9-10 points, of which at least 2 are cardinal features. Molecular testing for CdLS is indicated with a clinical score of 4-8 points.
***Major Criteria:*** Macrodontia (large teeth) Recurrent otitis media and/or hearing loss. Post-natal short stature (height <10th percentile) First-degree relative with KBG syndrome	***Cardinal features (2 points each of present):*** Synophrys and/or thick eyebrows Short nose, concave nasale ridge, and/or upturned nasal tip Hand oligodactyly and/or adactyly Long and/or smooth philtrum Thin upper lip vermilion and/or downturned corners of mouth Congenital diaphragmatic hernia
***Minor Criteria:*** Brachydactyly (short fingers) or hand anomaly Cryptorchidism (undescended testes) Seizures Feeding problems Palate abnormality Large anterior fontanelle and/or delayed closure Autism diagnosis	***Suggestive features (1 point each if present):*** Global developmental delay and/or intellectual disability Short fifth finger Prenatal growth retardation Small hands and/or feet Postnatal growth retardation Hirsutism

**Table 2:** Main clinical features of the described patient compared with MDR23, CdLS, and KBGS features.

Clinical Features of Our Patient	MDR23	CdLS	KBGS
Intellectual disability	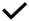	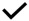	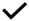
Delayed speech development	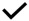		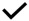
Hypotonia	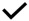		
Short stature and postnatal growth retardation	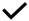	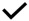	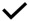
Dysmorphic feature	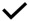	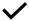	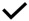
Macrodontia			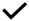
Synophrys		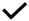	
Short nose and concave nasal ridge		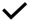	
Long philtrum		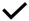	

**Table 3:** Characteristics of cases with CdLS, KBGS, and overlap syndrome treated with rhGH and height outcomes.

References	Case/Syndrome/and Additional Diseases	Genetic Variant	Age of Start and End of rHGh Therapy	Dose of rhGH	Height SDS at the Beginning of Treatment	Height SDS at the End of Treatment	Comments
**De Graaf et al.** [10]	Female patient born small for gestational age and with CdL syndrome	c.771+1G>A (chr5:36971139) in *NIPBL* gene	From 4.3 years to 8 years	0.86 mg/m^2^/day	−3.5 SDS	−1.4 SDS	rhGH therapy was indicated for small for gestational age. Increase in height SDS of 1.8 SDS following treatment with r-hGH
**Reynaert et al.** [11]	Male with KBG syndrome	c.3836del (p.Ser1279fs) in *ANKRD11* gene	From 10.5 years. Last evaluation when he was 13.8 years	35 μg/kg/day	−3.1 SDS	−1.6 SDS	Triptorelin treatment due to medical necessity was started at 12.5 years (height −1.7 SDS) because of the fast progression of puberty. At age 13.8 years triptorelin was stopped.
	Male with KBG Syndrome	c.1903_1907del (p.Lys635Glnf*26) in *ANKRD1* gene	From 7.4 years. Last evaluation when he was 12.4 years	30 μg/kg/day	−2.8 SDS	−0.7 SDS	The patient’s height increased by 1.0 SD during the first year on treatment and by another 1.1 SD in the subsequent 4 years.
**Xiu-Ying Ge et al.** [12]	Girl with Growth hormone deficiencies and KBG syndrome	c.2635 dupG, (p.Glu879fs) in *ANKRD11* gene	From 5.6 years to 7.6 years	50 μg/kg/day	−1.95 SDS	−0.70 SDS	rhGH therapy was indicated for Growth hormone deficiencies.
**Our patient**	Girl with overlap syndrome (MDR23, CdL and KBG syndrome)	c.890_891delTT (p.Leu297fs*15) in *SETD5* gene	From 12.6 years to 14.6 years	30 μg/kg/day	−5.22 SDS	−5.19 SDS *	After the first year of therapy, the growth rate had been 7.2 cm (+3.16 SDS). After 2 years of treatment, at the age of 14 years and 6 months, the growth rate had been 5.8 cm/year (+2.9 SDS).

* Height after two years of rhGH therapy. The therapy is not concluded, and the height is not definitive.

## Data Availability

The original contributions presented in this study are included in the article. Further inquiries can be directed to the corresponding author.
